# Molecular Mechanism of Long Noncoding RNA SNHG14 in Osteogenic Differentiation of Bone Marrow-Derived Mesenchymal Stem Cells through the NEDD4L/FOXA2/PCP4 Axis

**DOI:** 10.1155/2023/7545635

**Published:** 2023-01-05

**Authors:** Han Wang, Mingxing Fan, Yan An, Da He

**Affiliations:** Department of Spine Surgery, Beijing Jishuitan Hospital, The Fourth Medical College of Peking University, Beijing, China

## Abstract

Bone marrow-derived mesenchymal stem cells (BMSCs) have a superior potential of osteogenic differentiation (OD) and a promising stem cell type to treat bone defects. This study sought to investigate the molecular mechanism of long noncoding RNA small nucleolar RNA host gene 14 (SNHG14) in OD of BMSCs. Western blot analysis or RT-qPCR showed that SNHG14, neural precursor cell expressed developmentally downregulated 4-like (NEDD4L), and Purkinje cell protein 4 (PCP4) were upregulated whereas forkhead box A2 (FOXA2) was declined in OD of BMSCs. RT-qPCR and cell staining showed that SNHG14 downregulation repressed OD of BMSCs, as manifested by reductions in osteopontin and osteocalcin levels, the mineralization degree, and alkaline phosphatase activity. RNA/Co/chromatin immunoprecipitation and dual-luciferase assays and determination of mRNA stability and ubiquitination level showed that SNHG14 bound to human antigen R improves NEDD4L mRNA stability and expression, further promoted FOXA2 ubiquitination to inhibit FOXA2 expression, and then reduced FOXA2 enrichment on the PCP4 promoter to upregulate PCP4 transcription. Functional rescue experiments showed that the overexpression of NEDD4L or PCP4 and knockdown of FOXA2 both attenuated the inhibition of SNHG14 downregulation on OD of BMSCs. Overall, our findings suggested that SNHG14 promoted OD of BMSCs through the NEDD4L/FOXA2/PCP4 axis.

## 1. Introduction

Mesenchymal stem cells (MSCs) are nonhematopoietic, multipotent, and mature stem cells derived from different tissues and organs, such as the bone marrow, umbilical cord, peripheral blood, adipose tissue, brain, spleen, kidney, and liver [1, 2]. Bone marrow-derived MSCs (BMSCs) are the most widely used stem cells in cell therapy and tissue engineering owing to their properties in anti-inflammation, angiogenesis, organ repair, and bone regeneration [3–6]. Under the damage to osseous tissues, BMSCs can be differentiated into bone-forming osteoblasts to repair bone defects [7, 8]. Moreover, compared with other types of MSCs, such as adipose tissue MSCs, BMSCs show a superior potential for osteogenic differentiation (OD) *in vitro* as evidenced by more mineralized nodules, stronger alkaline phosphatase (ALP) activity, and higher levels of osteogenic specific genes [9]. However, the molecular mechanism in OD of BMSCs remains elusive and warrants further exploration.

Long noncoding RNA (lncRNAs), transcripts with a length of over 200 nucleotides, participate in OD of MSCs through multiple pathways and serve as promising therapeutic targets and prognostic parameters in the pathologic differentiation of MSCs [10, 11]. One such lncRNA, namely, small nucleolar RNA host gene 14 (SNHG14), is noted to play biological roles in cancers, inflammation, and organ damage [12–17]. It is also noteworthy that SNHG14 facilitates OD of MSCs by regulating microRNAs [18, 19]. However, relevant data on the molecular mechanism of SNHG14 in OD are still limited.

lncRNAs are known to bind to human antigen R (HuR) to regulate gene stability [20, 21]. The bioinformatics data revealed the binding of SNHG14 to HuR and HuR to neural precursor cells expressed developmentally downregulated 4-like (NEDD4L). NEDD4L is a member of the HECT domain-containing E3 ligase family that is associated with bone formation and a ubiquitin ligase for multiple proteins [22–25]. Precisely, NEDD4L is known to enhance the potential of MSCs in bone regeneration and repair [26]. Forkhead box protein A2 (FOXA2), belonging to the forkhead box transcription factor family responsible for bone metabolism and related diseases [27, 28], acts as a negative regulator of OD of BMSCs [29]. Inherently, FOXA2 binds to the gene promoter to negatively regulate gene expression [30, 31]. In addition, the bioinformatics data were indicative of the binding of FOXA2 to the promoter of Purkinje cell protein 4 (PCP4). PCP4 is not only a vital protein associated with Purkinje cell development, neurite outgrowth, and calcium homeostasis [32–34] but also a promoter of the cascade of OD and angiogenesis in BMSCs to alleviate osteoporosis [35]. Hence, whether the NEDD4L/FOXA2/PCP4 axis is involved in SNHG14-regulated OD of BMSCs should be addressed in this study.

In light of the aforementioned data, we deduced a hypothesis that SNHG14 plays a role in OD of BMSCs through HuR-mediated NEDD4L/FOXA2/PCP4 complex, which has not been mentioned in the previous study. Consequently, the current study was conducted to expound on the molecular mechanism by which SNHG14 induces OD of BMSCs and provide a novel theoretical reference for bone regeneration and repair.

## 2. Methods

### 2.1. Culture of Mouse BMSCs

Commercially available mouse BMSCs were obtained from the bone marrow of Balb/c mice (MUCMX-01001, Cyagen Biosciences, Guangzhou, China) and cultured in *α*-modified Eagle medium (Life Technologies, Carlsbad, CA, USA) supplemented with 10% fetal bovine serum (FBS, Life Technologies), 100 U/mL penicillin (Sigma, St. Louis, MO, USA), and 100 mg/mL streptomycin (Sigma). Cells were cultured under the condition of 37°C and 5% CO_2_. The culture medium was changed every two days. The third generation of BMSCs was adopted for experimentation.

### 2.2. Characterization of BMSCs

BMSCs (5 × 10^5^) were detached by 0.25% trypsin containing 0.53 mM ethylenediaminetetraacetic acid and added with FBS to terminate the reaction. After three washes with phosphate-buffered saline (PBS), BMSCs were cultured in fluorescence-activated cell sorting (FACS) buffer (BD Biosciences) containing PBS and incubated with antibodies CD90 (1 : 200, ab25672, Abcam, Cambridge, MA, USA), CD105 (1 : 200, ab184667, Abcam), CD34 (1 : 200, ab23830, Abcam), and CD117 (1 : 200, ab210244, Abcam) for 30 min. After another 3 washes with PBS, the positive rates of surface markers were determined by BD Influx cell sorter (BD Biosciences, San Jose, CA, USA).

BMSCs were cultured and induced with osteogenic or adipogenic differentiation. BMSCs were loaded in 12-well plates at a concentration of 1.5 × 10^4^ cells/cm^2^ and cultured in an OD culture medium for 14 d and treated with alizarin red staining (ARS) and ALP staining to quantify the potential of OD. BMSCs (1.5 × 10^4^ cells/cm^2^) were seeded in adipogenic differentiation (AD) culture medium [Dulbecco's modified Eagle medium (DMEM), 10% FBS, 1 *μ*M dexamethasone, 10 *μ*g/mL insulin (Sigma), 0.5 mM 3-isobutyl-1-methylxanthine (Sigma), and 0.2 mM indomethacin (Sigma)]. The culture medium was changed every three days. After 14 d, BMSCs were fixed with 4% paraformaldehyde by oil red O staining for 15 min, washed thrice with PBS, and stained with oil red O working solution for 15 min. The stained cells were photographed by a microscope to quantify the potential of adipogenic differentiation.

### 2.3. Osteogenic Induction (OI) and Treatment of BMSCs

When BMSCs of the third generation reached 60-70% confluence, cells were treated with OI using an OI culture medium [DMEM, 10% FBS, 50 *μ*g/mL L-ascorbic acid (Sigma, Shanghai, China), 10 mM *β*-glycerophosphoric acid (Sigma), 0.1 *μ*M dexamethasone (Sigma), 100 U/mL penicillin, and 100 *μ*g/mL streptomycin]. The culture medium was changed every three days. Different kinds of analyses were performed at 5 induction time points (0 d, 3 d, 7 d, 10 d, and 14 d).

Lentiviral vectors containing short hairpin RNA targeting SNHG14 (sh-SNHG14), overexpression plasmids of NEDD4L (oe-NEDD4L), sh-HuR, sh-FOXA2, and oe-PCP4 and their negative controls were provided by GenePharma (Shanghai, China). As for infection, BMSCs were incubated with lentivirus particles (10^9^ TU/mL) and polybrene (5 *μ*g/mL) in the culture medium. After 24 h, the infected culture medium was discarded. After 3 d, cells were screened with puromycin (4 *μ*g/mL) for the subsequent experiments. The efficiency of lentiviral infection was determined by quantitative polymerase chain reaction (qPCR) and Western blot analysis.

### 2.4. Alizarin Red Staining

Mineral deposition in BMSCs was observed by ARS after OI. Cells were treated with 4% paraformaldehyde at room temperature for 15 min, washed with PBS three times, and stained with alizarin red (10 *μ*L/mL, Sigma) at room temperature for 30 min. The images of stained cells were captured by a microscope. For ARS quantification, cells were incubated with 10% cetylpyridinium chloride monohydrate (Sigma) for 30 min, gently shaken, and rinsed with PBS. The solution was collected and the absorbance at a wavelength of 562 nM was determined using a microplate reader.

### 2.5. ALP Activity and ALP Staining

To determine ALP activity, BMSCs were lysed using the radioimmunoprecipitation method (RIPA, Beyotime, Shanghai, China), and APL activity was determined using an APL activity assay kit (Beyotime). Briefly, 10 *μ*L of lysis solution was incubated with 90 *μ*L of fresh solution at 37°C for 30 min and added with 100 *μ*L 0.5 N NaOH to terminate the reaction. For ALP staining, BMSCs were treated with 4% paraformaldehyde at room temperature for 15 min, washed thrice with PBS, and stained using BCIP/NBT ALP Color Development kit (Beyotime).

### 2.6. Subcellular Fractionation Assay

RNA was separated from cytoplasmic or nuclear components of BMSCs using PARIS kits (Invitrogen). With GAPDH and U6 as the controls, SNHG14 expression levels in both components were determined using RT-qPCR.

### 2.7. RNA Fluorescence *In Situ* Hybridization (FISH)

Cy3-labelled SNHG14 probe was synthesized by Guangzhou RiboBio Co., Ltd. (Guangzhou, China). BMSCs were fixed with 4% paraformaldehyde and hybridized with a hybridization solution containing the SNHG14 probe at 37°C in the dark overnight, and then nuclei were stained with 4,6-diamino-2-phenylindole (Solaribo, Beijing, China). All images were obtained with the help of DMi8 microscope (Leica Microsystems, Mannheim, Germany).

### 2.8. Bioinformatics Analysis

The binding scores of HuR and SNHG14 or NEDD4L were predicted on the RNA-Protein Interaction Prediction (RPISeq) website (http://pridb.gdcb.iastate.edu/RPISeq/) [36]. The binding sequence of FOXA2 and the PCP4 promoter was predicted on the JASPAR database (https://jaspar.genereg.net/) [37].

### 2.9. RNA Immunoprecipitation (RIP) Assay

RIP assay was conducted according to the protocol of Magna RIP assay kits (MilliporeSigma, Bedford, MA, USA). BMSCs were lysed and incubated with magnetic beads preconjugated with the antibody anti-HuR (ab200342, Abcam) or anti-IgG (ab172730, Abcam) to capture RNA compounds. Bound RNA compounds were eluted and separated for RT-qPCR analysis.

### 2.10. mRNA Stability Assay

The mRNA stability of NEDD4L was determined by actinomycin D (ActD, Sigma). Simply put, BMSC culture medium was added with 5 *μ*g/mL ActD. After 0, 2, 4, and 6 h, RNA was separated from cells, and the mRNA level of NEDD4L was determined by RT-qPCR.

### 2.11. Coimmunoprecipitation Assay

BMSCs were lysed in the lysis buffer. Then, the lysis solution of BMSCs was incubated with antibodies anti-NEDD4L and anti-IgG (ab172730, Abcam) at 4°C overnight. Next, the cocktail was incubated with protein A/G agarose beads (Novex, Oslo, Norway) at 4°C for 3 h. After that, beads were collected, washed, resuspended, and boiled and proteins were separated by SDS-PAGE and transferred to polyvinylidene difluoride membranes, followed by Western blot analysis.

### 2.12. Ubiquitination Assay

*In vitro* ubiquitination assay was performed in BMSCs. After lentiviral infection, BMSCs were treated with 40 *μ*M MG132 for 6 h and lysed. The collected lysis solution was incubated with protein A/G agarose containing antibodies anti-FOXA2 (ab256493, Abcam) and anti-IgG (ab172730, Abcam) at 4°C overnight. Then, Western blot analysis was performed using an antiubiquitin antibody (ab19247, Abcam).

### 2.13. Dual-Luciferase Assay

According to the binding sites of FOXA2 and the PCP4 promoter from the JASPAR database, the relevant sequence was amplified and cloned into pGL3 control vectors (Promega, Madison, WI, USA) to construct the PCP4 wild-type vector (PCP4 WT), and the mutant sequence of PCP4 was cloned into pGL3 to construct PCP4 mutant-type vector (PCP4 MUT). Then, BMSCs were cotreated with the above-constructed plasmids and sh-FOXA2 or sh-NC. The luciferase activity was analyzed using the dual-luciferase report and analysis system (Promega).

### 2.14. Chromatin Immunoprecipitation (ChIP)

BMSCs were treated with 4% methanal to produce DNA-protein crosslink. Then, the cell lysis was ultrasonically processed and immunoprecipitated using the antibodies of FOXA2 (ab256493, Abcam) or IgG (ab172730, Abcam). Immunoprecipitated chromatin DNA was eluted, decrosslinked, and purified, followed by RT-qPCR analysis. Primers used in ChIP were as follows: forward primer: 5′-ATTGAGCCTTCATAAGGGCG-3′, reverse primer: 5′-GTATGCCCCTATATGTATG-3′.

### 2.15. qPCR

RNA was extracted from BMSCs using the TRIzol reagent (Sigma). Then, 1 *μ*g total RNA was reverse-transcribed using RevertAid First Strand cDNA Synthesis kit (Fermentas, Shanghai, China). RT-qPCR was performed using LightCycler 480 SYBR Green qPCR Supermix (Roche, Shanghai, China) on Roche LightCycler 480 II real-time PCR detection system. With GAPDH as the internal control, primers are listed in Table 1, and the relative gene expression was calculated using the 2^−ΔΔCt^ method [38].

### 2.16. Western Blot Analysis

Protein extracts of BMSCs were prepared with RIPA lysis buffer containing proteasome inhibitor (Beyotime). The total protein was separated using 10% SDS-PAGE and transferred to polyvinylidene difluoride membranes. After 1 h of blockade with 5% bovine serum albumin, membranes were incubated with antibodies OPN (ab283656, 1 : 1000), OCN (ab93876, 1 : 100), NEDD4L (ab245522, 1 : 2000), HuR (ab200342, 1 : 1000), FOXA2 (ab256493, 1 : 1000), and *β*-actin (ab8227, 1 : 1000) at 4°C overnight and with the secondary antibody (ab205718, 1 : 2000) for 1 h. Immunoreactive bands were observed using the enhanced chemiluminescence assay reagent (Millipore), and the grayscale value was quantified using a chemiluminescence determination system (Bio-Rad, Hercules, CA, USA). All antibodies were provided by Abcam.

### 2.17. Statistical Analysis

All data were processed with SPSS 21.0 (IBM Corp., Armonk, NY, USA) and GraphPad Prism 8.0 (GraphPad Software Inc., San Diego, CA, USA) and conformed to normal distribution and homogeneity of variance. Pairwise comparisons were analyzed using the *t*-test, and multigroup comparisons were analyzed using one-way or two-way analysis of variance (ANOVA), followed by Tukey's multiple comparison test. A value of *P* < 0.05 referred to statistical significance and a value of *P* < 0.01 referred to highly statistical significance.

## 3. Results

### 3.1. Characterization of Mouse BMSCs

**Figure 1:**
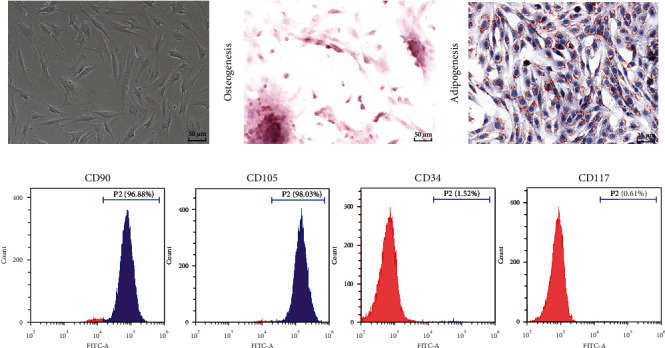
Characterization of mouse BMSCs. (a) BMSCs were observed and photographed under an inverted microscope. (b, c) Staining with alizarin red/oil red O after osteogenic/adipogenic induction. (d) Expression levels of CD90, CD105, CD34, and CD117 on the surface of BMSCs were determined by flow cytometry. Cell experiments were repeated 3 times.

BMSCs have a great potential for OD and are considered the most promising cell type for bone regeneration and repair in tissue engineering [39, 40]. Then, we cultured BMSCs, and the microscopy showed that BMSCs were spindle-shaped (Figure 1(a)) and showed strong potential for OD and AD (Figures 1(b) and 1(c)). The analysis of surface biomarkers of BMSCs showed that BMSCs were positive for CD90 and CD105 while negative for CD34 and CD117 (Figure 1(d)). The above results suggested that we characterized BMSCs successfully.

### 3.2. SNHG14 Downregulation Represses OD of BMSCs

**Figure 2:**
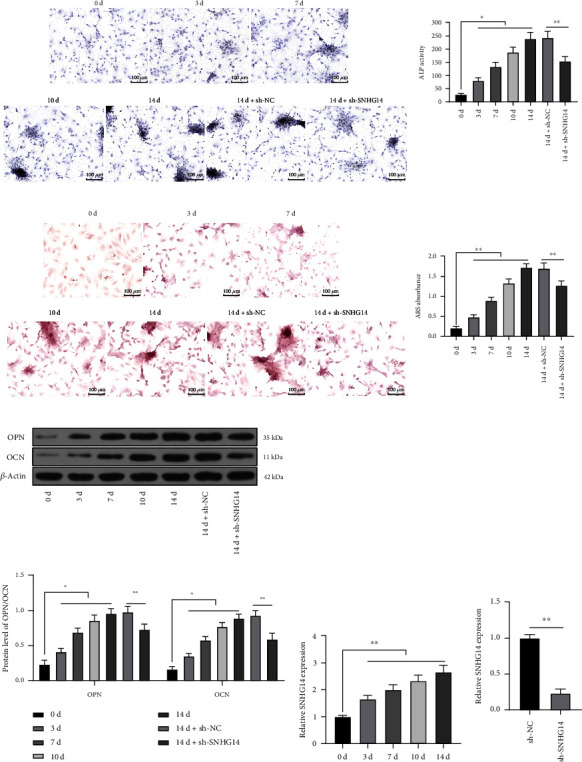
SNHG14 downregulation represses OD of BMSCs. BMSCs were treated with osteogenic induction (OI) and infected with SNHG14 shRNA lentivirus (sh-SNHG14), with NC shRNA (sh-NC) as the control. (a) Alkaline phosphatase (ALP) staining and ALP activity determination. (b) Alizarin red staining and determination of mineralized nodules. (c) expression levels of OCN and OPN were determined by Western blot analysis. (d–e) SNHG14 expression level was determined by RT-qPCR. Cell experiments were repeated 3 times. Data were represented as mean ± standard deviation. Multigroup comparisons in panels (a), (b), and (d) were analyzed using one-way ANOVA, and multigroup comparisons in panel (c) were analyzed by two-way ANOVA, followed by Tukey's multiple comparison test; pairwise comparisons in panel (e) were analyzed using the *t*-test. ^∗^*P* < 0.05, ^∗∗^*P* < 0.01.

lncRNA SNHG14 has been documented to be overexpressed in OD [18, 19]. BMSCs were treated with OI at different time points and showed increases in ALP activity, degree of mineralization (*P* < 0.05, Figures 2(a) and 2(b)), and expression levels of OPN and OCN (*P* < 0.05, Figure 2(c)). It was also found that SNHG14 expression level was increased in a time-dependent manner (*P* < 0.01, Figure 2(d)). BMSCs with the highest expression level of SNHG14 on 14 d of OI were used for the subsequent experiments. To explore the impact of SNHG14 on OD of BMSCs, BMSCs were infected with SNHG14 shRNA lentivirus (sh-SNHG14) to downregulate intercellular SNHG14 expression (*P* < 0.01, Figure 2(e)). Our results showed that after SNHG14 downregulation, ALP activity and the degree of mineralization in BMSCs were reduced (*P* < 0.01, Figures 2(a) and 2(b)) and expression levels of OPN and OCN were also declined (*P* < 0.01, Figure 2(c)). These results suggested that SNHG14 downregulation repressed OD of BMSCs.

### 3.3. SNHG14 Binds to HuR to Stabilize NEDD4L Expression

**Figure 3:**
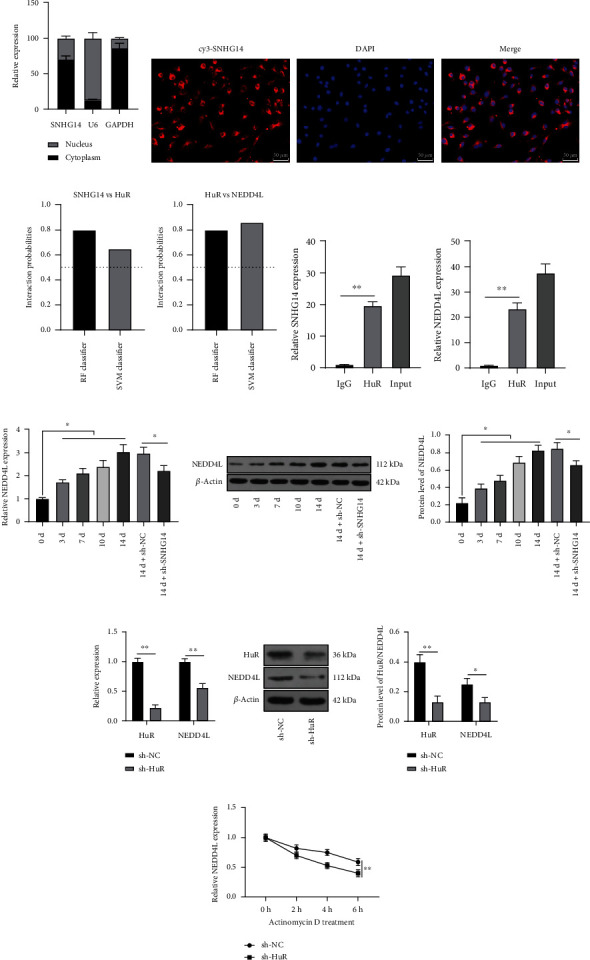
SNHG14 binds to HuR to stabilize NEDD4L expression. (a, b) Subcellular localization of SNHG14 was analyzed by the subcellular fractionation assay and RNA FISH. (c) Binding probabilities of SNHG14 and HuR, HuR and NEDD4L were predicted on the RPISeq database. (d) Bindings of SNHG14 to HuR and HuR to NEDD4L were analyzed by RIP assay. (e, f) NEDD4L expression level was determined by RT-qPCR and Western blot analysis; BMSCs were infected with HuR shRNA lentivirus (sh-HuR), with sh-NC as the negative control, (g, h) Expression levels of HuR and NEDD4L were determined by RT-qPCR and Western blot analysis. (i) Half-life period of NEDD4L was determined by RT-qPCR. Cell experiments were repeated 3 times. Data were represented as mean ± standard deviation. Multigroup comparisons in panels (d)–(f) were analyzed using one-way ANOVA, and pairwise comparisons in panels (g)–(i) were analyzed using two-way ANOVA, followed by Tukey's multiple comparison test. ^∗^*P* < 0.05, ^∗∗^*P* < 0.01.

To explore the downstream mechanism of SNHG14, we found that SNHG14 was mainly located in the cytoplasm of BMSCs (Figures 3(a) and 3(b)). It has been reported that the cytoplasmic lncRNA can bind to HuR to regulate gene stability [20]. The RPISeq website predicted high probabilities of the binding of SNHG14 to HuR and HuR to NEDD4L (Figure 3(c)). NEDD4L has been shown to be upregulated in the process of OD [26]. RIP assay validated binding relationships between SNHG14 and HuR and between HuR and NEDD4L (*P* < 0.01, Figure 3(d)). In addition, NEDD4L expression was increased in BMSCs in a time-dependent manner after OI and was decreased after SNHG14 downregulation (*P* < 0.05, Figures 3(e) and 3(f)). To validate whether SNHG14 stabilizes NEDD4L expression by binding to HuR, HuR was downregulated in BMSCs by infection with HuR shRNA lentivirus (sh-HuR), upon which NEDD4L expression level was reduced accordingly (*P* < 0.01, Figures 3(g) and 3(h)) and the mRNA stability of NEDD4L was also significantly declined (*P* < 0.01, Figure 3(i)). The above results suggested that SNHG14 bound to HuR to stabilize NEDD4L expression.

### 3.4. NEDD4L Overexpression Attenuates the Inhibition of SNHG14 Downregulation on OD of BMSCs

**Figure 4:**
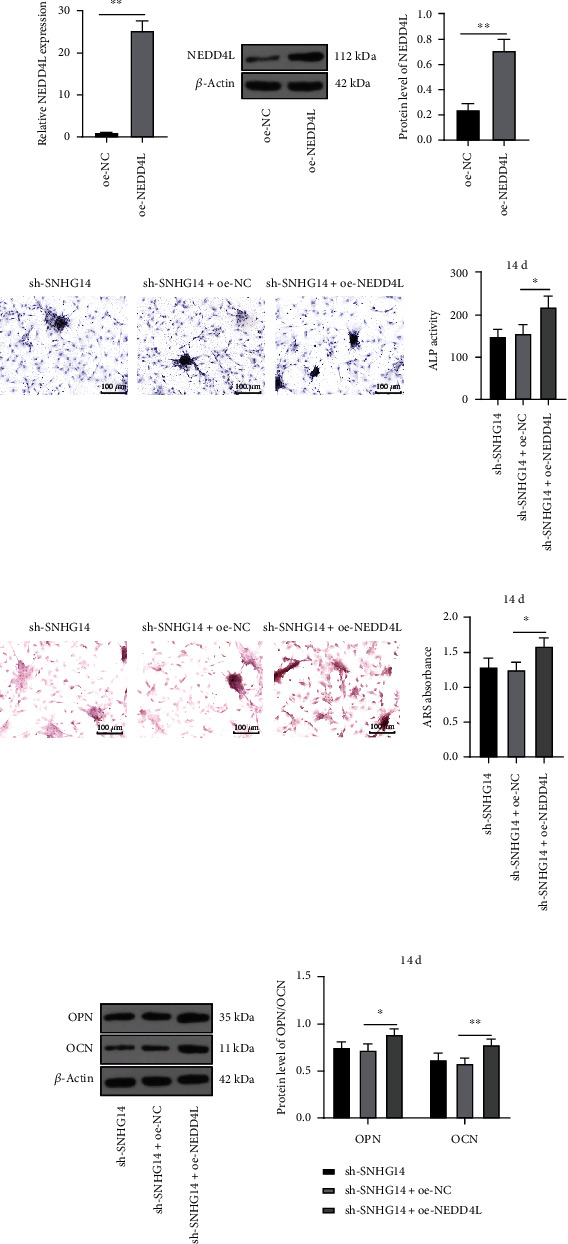
NEDD4L overexpression attenuates the inhibition of SNHG14 downregulation on OD of BMSCs. BMSCs were infected with overexpression plasmid of NEDD4L lentivirus (oe-NEDD4L), with oe-NC as the control. (a, b) NEDD4L expression level was determined by RT-qPCR and Western blot analysis. (c) ALP staining and ALP activity determination. (d) Alizarin red staining and determination of mineralized nodules. (e) Expression levels of OCN and OPN were determined by Western blot analysis. Cell experiments were repeated 3 times. Data were represented as mean ± standard deviation. Multigroup comparisons in panels (c) and (d) were analyzed using one-way ANOVA, and in panel (e) were analyzed using two-way ANOVA, followed by Tukey's multiple comparison test. Pairwise comparisons in panels (a) and (b) were analyzed using the *t*-test. ^∗^*P* < 0.05, ^∗∗^*P* < 0.01.

Next, BMSCs were infected with overexpression plasmid of NEDD4L lentivirus (oe-NEDD4L) to upregulate NEDD4L expression (*P* < 0.01, Figures 4(a) and 4(b)), followed by the combined treatment with sh-SNHG14 to explore the impact of NEDD4L on SNHG14-regulated OD of BMSCs. After the treatment of oe-NEDD4L and OI (14 d), ALP activity and mineralization degree were enhanced (*P* < 0.05, Figures 4(c) and 4(d)) and expression levels of OPN and OCN were augmented (*P* < 0.05, Figure 4(e)). The above results suggested that NEDD4L overexpression attenuated the inhibition of SNHG14 downregulation on OD of BMSCs.

### 3.5. NEDD4L Inhibits FOXA2 Expression through Ubiquitination Modification and Promotes PCP4 Transcription

**Figure 5:**
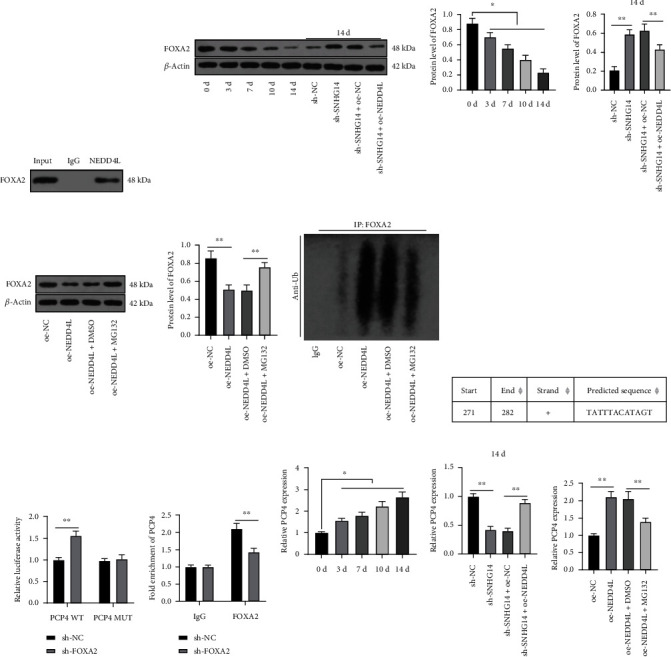
NEDD4L inhibits FOXA2 expression through ubiquitination modification and promotes PCP4 transcription. (a) Binding of NEDD4L to FOXA2 was examined by coimmunoprecipitation. (b) FOXA2 expression level after osteogenic induction (OI) was determined by Western blot analysis. (c) FOXA2 expression level in BMSCs was determined by Western blot analysis. (d) Ubiquitination level of FOXA2 was determined by the ubiquitination assay. (e) Binding of FOXA2 to the PCP4 promoter was predicted on the JASPAR database. (f, g) FOXA2 to the PCP4 promoter was analyzed by dual-luciferase and ChIP assays. (h, i) mRNA level of PCP4 after OI and in BMSCs was determined by RT-qPCR. Cell experiments were repeated 3 times. Data were represented as mean ± standard deviation. Multigroup comparisons in panels (b) and (c) and (h) and (i) were analyzed using one-way ANOVA and in panels (f) and (g) were analyzed using two-way ANOVA, followed by Tukey's multiple comparison test. ^∗^*P* < 0.05, ^∗∗^*P* < 0.01.

NEDD4L regulates the levels of proteins through ubiquitination modification. FOXA2 has been reported to be poorly expressed in OD and modified by ubiquitination [29, 41]. Coimmunoprecipitation showed that NEDD4L can bind to FOXA2 (Figure 5(a)). Western blot analysis showed that the protein level of FOXA2 was decreased after OI of BMSCs and increased after SNHG14 downregulation (*P* < 0.05, Figure 5(b)), and after NEDD4L overexpression, FOXA2 protein level was decreased (*P* < 0.01, Figures 5(b) and 5(c)) and the ubiquitination level of FOAX2 was elevated (Figure 5(d)). After the treatment of MG132, the protein level of FOXA2 was elevated and the ubiquitination level of FOAX2 was reduced (*P* < 0.01, Figures 5(c) and 5(d)). Transcription factor FOXA2 can bind to the gene promoter to repress gene expression [30], and PCP4 has been demonstrated to be highly expressed in OD [35]. The JASPAR database unveiled that FOXA2 can bind to the PCP4 promoter (Figure 5(e)). The dual-luciferase assay showed a binding relationship between FOXA2 and the PCP4 promoter, which was further validated by ChIP assay (*P* < 0.01, Figures 5(f) and 5(g)). RT-qPCR showed that PCP4 transcriptional level was increased after OI of BMSCs, declined after SNHG14 downregulation, and augmented after NEDD4L overexpression (*P* < 0.05, Figures 5(h) and 5(i)). In addition, with the increase in FOXA2 protein level, PCP4 transcriptional level was decreased (*P* < 0.01, Figure 5(i)). The above results suggested that NEDD4L inhibited FOXA2 expression through ubiquitination modification and promoted PCP4 transcription.

### 3.6. FOXA2 Downregulation Attenuates the Inhibition of Silencing SNHG14 on OD of BMSCs

**Figure 6:**
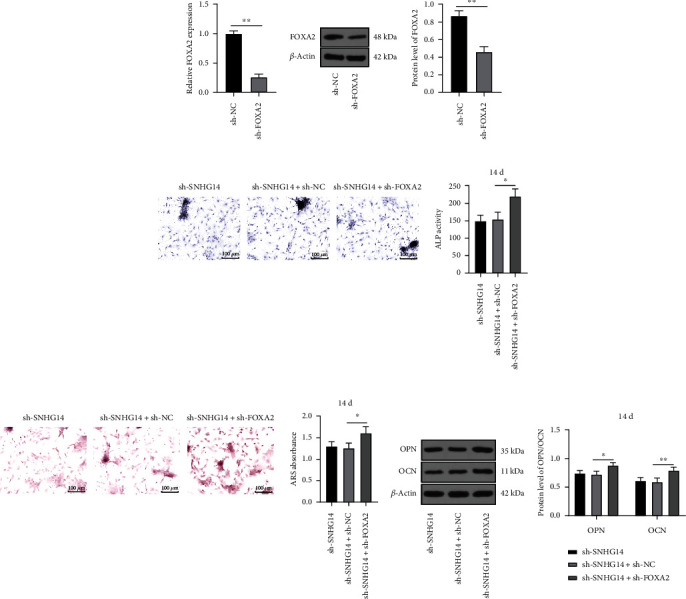
FOXA2 downregulation attenuates the inhibition of silencing SNHG14 on OD of BMSCs. BMSCs were infected with FOXA2 shRNA lentivirus (sh-FOXA2), with sh-NC as the control. (a, b) FOXA2 expression level was determined by RT-qPCR and Western blot analysis. (c) ALP staining and ALP activity determination. (d) Alizarin red staining and determination of mineralized nodules. (e) Expression levels of OCN and OPN were determined by Western blot analysis. Cell experiments were repeated 3 times. Data were represented as mean ± standard deviation. Multigroup comparisons in panels (c) and (d) were analyzed using one-way ANOVA and in panel (e) were analyzed using two-way ANOVA, followed by Tukey's multiple comparison test. Pairwise comparisons in panels (a) and (b) were analyzed using the *t*-test. ^∗^*P* < 0.05, ^∗∗^*P* < 0.01.

FOXA2 expression in BMSCs was inhibited by infection with FOXA2 shRNA lentivirus (sh-FOXA2) (*P* < 0.01, Figures 6(a) and 6(b)), followed by the combined treatment with sh-SNHG14. Our results showed that after knockdown of FOXA2, ALP activity and mineralization degree were augmented (*P* < 0.05, Figures 6(c) and 6(d)) and expression levels of OPN and OCN were increased (*P* < 0.05, Figure 6(e)). The above results suggested that FOXA2 downregulation attenuated the inhibition of silencing SNHG14 on OD of BMSCs.

### 3.7. PCP4 Overexpression Attenuates the Inhibition of Silencing SNHG14 on OD of BMSCs

**Figure 7:**
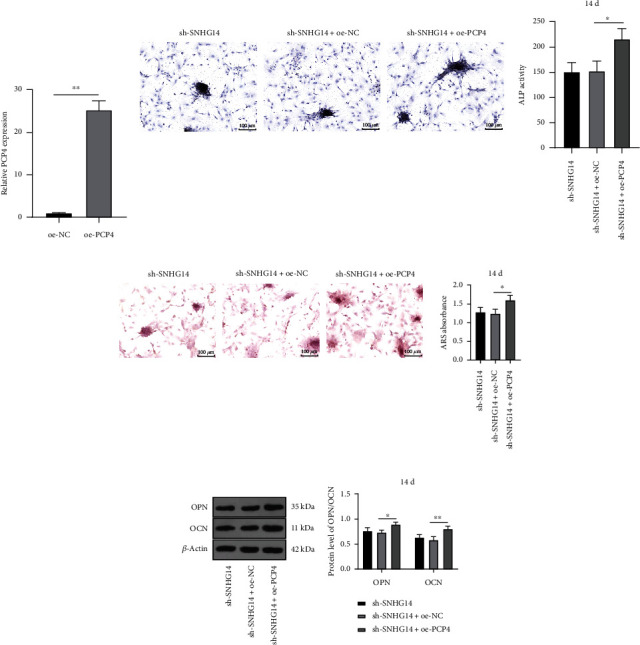
PCP4 overexpression attenuates the inhibition of silencing SNHG14 on OD of BMSCs. BMSCs were infected with overexpression plasmid of PCP4 lentivirus (oe-PCP4), with oe-NC as the control. (a) mRNA level of PCP4 was determined by RT-qPCR. (b) ALP staining and ALP activity determination. (c) Alizarin red staining and determination of mineralized nodules. (d) Expression levels of OCN and OPN were determined by Western blot analysis. Cell experiments were repeated 3 times. Data were represented as mean ± standard deviation. Multigroup comparisons in panels (b) and (c) were analyzed using one-way ANOVA and in panel (d) were analyzed using two-way ANOVA, followed by Tukey's multiple comparison test. Pairwise comparisons in panel (a) were analyzed using the *t*-test. ^∗^*P* < 0.05, ^∗∗^*P* < 0.01.

At last, to explore the impact of PCP4 transcription on OD of BMSCs, BMSCs were infected with overexpression plasmid of PCP4 lentivirus (oe-PCP4) to upregulate PCP4 transcriptional level (*P* < 0.01, Figure 7(a)). Our results elicited that, relative to the si-SNHG14 group, the OD potential of BMSCs was enhanced in the si-SNHG14+oe-PCP4 group (*P* < 0.05, Figures 7(b)–7(d)). The above results suggested that PCP4 overexpression may attenuate the inhibition of silencing SNHG14 on OD of BMSCs.

## 4. Discussion

**Figure 8:**
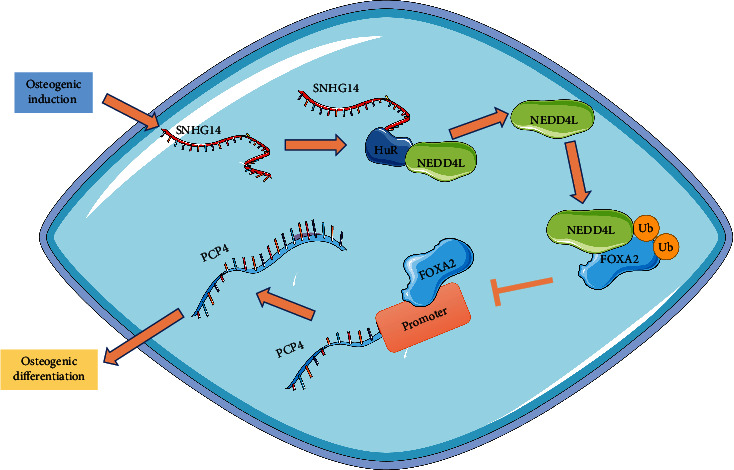
Mechanism of SNHG14 in OD of BMSCs. Osteogenic induction upregulated SNHG14 expression level in BMSCs, and SNHG14 bound to HuR increases NEDD4L expression, promoted ubiquitination of FOXA2 to inhibit FOXA2 expression, reduced the enrichment level of FOXA2 on the PCP4 promoter, and upregulated PCP4 transcriptional level, consequently promoting OD of BMSCs.

OD of BMSCs is effective for bone regeneration and healing fractures and osteoporosis, and this process is correlated with various molecular genetic mechanisms and signaling pathways [42–44]. Accumulating evidence has denoted that lncRNAs play epigenetic roles in OD of BMSCs and relevant diseases [45]. In the present study, our findings uncovered that SNHG14 promoted OD of BMSCs through the NEDD4L/FOXA2/PCP4 axis (Figure 8).

SNHG14 is known to be declined in BMSCs from patients with osteoporosis, and it promotes OD of BMSCs by targeting the miR-185-5p/WISP2 axis [19]. Likewise, inhibition of SNHG14 can block BMSCs to differentiate into osteoblasts by targeting miR-2861 [18]. ALP is enriched in cells of mineralized tissues and is a positive indicator of osteoblast differentiation, and OPN and OCN are also critical osteoblast-specific genes that indicate the presence of osteoblasts [46, 47]. Accordingly, our results suggested that SNHG14 was increased after OI of BMSCs in a time-independent manner and sh-SNHG14 repressed OD of BMSCs as manifested by reductions in ALP activity, mineralization degree, and levels of OPN and OCN. To explore the downstream mechanism of SNHG14, we unveiled cytoplasmic localization of SNHG14, suggesting its possibility of binding to HuR and further stabilizing downstream gene expression [20, 21]. NEDD4L is noted to accelerate the proliferation and differentiation of immature osteoblasts by removing C-terminal phosphorylated Smad1 and then enhance skeletogenesis [48, 49]. Similarly, WWP2, a member of the NEDD4 family, is potent to increase the transcriptional activity of runt-related transcription factor 2 (Runx2) by inducing monoubiquitination to enhance osteogenesis [50]. Specifically, NEDD4L can accelerate OD of MSCs through activation of the Akt pathway [26] and enhance OD of BMSCs through inhibition of Hippo signaling [51] and degradation of the p38 MAPK pathway [52]. In our study, our experiments revealed that NEDD4L expression was enhanced after OI of BMSCs in a time-dependent manner and SNHG14 bound to HuR stabilizes NEDD4L expression, and NEDD4L overexpression attenuated the inhibitory role of SNHG14 downregulation in OD of BMSCs. Altogether, our findings initially demonstrated that SNHG14 facilitated OD of BMSCs through HuR-mediated upregulation of NEDD4L.

NEDD4L as an E3 ubiquitin-protein ligase mediates ubiquitination modification to regulate the protein level [53, 54], and our bioinformatics data suggested FOXA2 was a target of NEDD4L. FOX family members are essential for bone metabolism and function as regulators of OD of MSCs [28, 55–57]. FOXA2 is identified as a critical transcription factor involved in the process of MSCs in the repair of the injured pancreas, intestinal and hepatic tissues, and neurons [58–61]. Interestingly, silencing FOXA2 is shown to promote OD of BMSCs and bone healing through activation of the ERK signaling pathway [29]. Besides, FOXA2 as a transcription factor can bind to the gene promoter to repress downstream gene expression [30, 31], and our bioinformatics data demonstrated that PCP4 was a target of FOXA2. In previous studies, PCP4 mRNA level is upregulated in OD of BMSCs [62]; PCP4 potentiates proliferation and differentiation of osteoblasts by blocking the c-Jun NH_2_-terminal kinase signaling pathway [63]; PCP4 knockdown suppresses OD of BMSCs in the progression of osteoporosis [35]; mechanically, PCP4 serves as a positive regulator of proosteogenic Runx2 in odontoblast differentiation of root progenitor cells [64]. Our subsequent experimental data revealed that FOXA2 protein level was decreased and PCP4 transcriptional level was increased after OI of BMSCs and NEDD4L inhibited FOXA2 expression through ubiquitin modification and further promoted PCP4 transcription. In functional rescue experiments, knockdown of FOXA2 or overexpression of PCP4 attenuated the inhibitory role of silencing SNHG14 in OD of BMSCs. Collectively, our findings identified a novel mechanism of SNHG14 in OD of BMSCs wherein SNHG14 induces HuR-mediated upregulation of NEDD4L to repress FOXA2 expression through ubiquitination modification and upregulate PCP4 transcription, thereby facilitating OD of BMSCs.

## 5. Conclusion

To conclude, our study was the first of its kind to unveil the molecular mechanism of SNHG14 in OD of BMSCs through the NEDD4L/FOXA2/PCP4 axis and may facilitate the clinical study of SNHG14 in bone regeneration and repair. However, the cause of SNHG14 alteration after OD of BMSCs, whether other ubiquitin enzymes and transcription factors participate in the regulation of SNHG14 in OD of BMSCs, and changes in PCP4 protein levels in BMSCs remain unknown. Besides, our study failed to validate the SNHG14-HuR-NEDD4L-FOXA2-PCP4 mechanism in animal models. In the next step, we shall explore the upstream mechanism of SNHG14 in OD of BMSCs and other ubiquitin enzymes and transcription factors in SNHG14-regulated OD of BMSCs and select more types of BMSCs to validate our mechanism, so as to provide updating theoretical knowledge of bone regeneration and repair.

## Figures and Tables

**Table 1:** PCR.

Gene	Sequence (5′-3′)
SNHG14	F: CATCTGTGTGGGGCCTTATGA
	R: GAGCCTCCTGTTTACCAACCT
NEDD4L	F: TTGACCTCGCCAAGAAGGAC
	R: TTCCACTTTGGGTTCAGCGT
HuR	F: CACCACCAGGCACAGAGATT
	R: CGGGGACATTGACACCAGAA
PCP4	F: GTGAGAGACAAAGTGCCGGAG
	R: TGGACTTTCTTCTGCCCATCA
FOXA2	F: ATGCACTCGGCTTCCAGTAT
	R: CTCACGGAAGAGTAGCCCTC
GAPDH	F: GGTCCCAGCTTAGGTTCATCA
	R: AATCCGTTCACACCGACCTT

## Data Availability

The datasets generated during and/or analyzed during the current study are available from the corresponding author on reasonable request.
